# Soybean (*Glycine max* L. Merr.) seedlings response to shading: leaf structure, photosynthesis and proteomic analysis

**DOI:** 10.1186/s12870-019-1633-1

**Published:** 2019-01-21

**Authors:** Yuanfang Fan, Junxu Chen, Zhonglin Wang, Tingting Tan, Shenglan Li, Jiafeng Li, Beibei Wang, Jiawei Zhang, Yajiao Cheng, Xiaoling Wu, Wenyu Yang, Feng Yang

**Affiliations:** 1 College of Agronomy, Sichuan Agricultural University, Huimin Road 211, Wenjiang District, Chengdu, 611130 People’s Republic of China; 2 Sichuan Engineering Research Center for Crop Strip Intercropping System, Chengdu, 611130 People’s Republic of China; 3 Key Laboratory of Crop Ecophysiology and Farming System in Southwest, Ministry of Agriculture, Chengdu, 611130 People’s Republic of China

**Keywords:** Soybean, Shading, Leaf structure, Photosynthesis, iTRAQ, Proteomics

## Abstract

**Background:**

Intercropping and close planting are important cultivation methods that increase soybean yield in agricultural production. However, plant shading is a major abiotic stress factor that influences soybean growth and development. Although shade affects leaf morphological parameters and decreases leaf photosynthesis capacity, information on the responses of soybean leaf photosynthesis to shading at proteomic level is still lacking.

**Results:**

Compared with leaves under normal light (CK) treatment, leaves under shading treatment exhibited decreased palisade and spongy tissue thicknesses but significantly increased cell gap. Although shade increased the number of the chloroplast, the thickness of the grana lamella and the photosynthetic pigments per unit mass, but the size of the chloroplast and starch grains and the rate of net photosynthesis decreased compared with those of under CK treatment. A total of 248 differentially expressed proteins, among which 138 were upregulated, and 110 were downregulated, in soybean leaves under shading and CK treatments were detected via isobaric tags for relative and absolute quantification labeling in the three biological repeats. Differentially expressed proteins were classified into 3 large and 20 small groups. Most proteins involved in porphyrin and chlorophyll metabolism, photosynthesis-antenna proteins and carbon fixation in photosynthetic organisms were upregulated. By contrast, proteins involved in photosynthesis were downregulated. The gene family members corresponding to differentially expressed proteins, including protochlorophyllide reductase (Glyma06g247100), geranylgeranyl hydrogenase (Ggh), LHCB1 (Lhcb1) and ferredoxin (*N/A*) involved in the porphyrin and chlorophyll metabolism, photosynthesis-antenna proteins and photosynthesis pathway were verified with real-time qPCR. The results showed that the expression patterns of the genes were consistent with the expression patterns of the corresponding proteins.

**Conclusions:**

This study combined the variation of the soybean leaf structure and differentially expressed proteins of soybean leaves under shading. These results demonstrated that shade condition increased the light capture efficiency of photosystem II (PSII) in soybean leaves but decreased the capacity from PSII transmitted to photosystem II (PSI). This maybe the major reason that the photosynthetic capacity was decreased in shading.

**Electronic supplementary material:**

The online version of this article (10.1186/s12870-019-1633-1) contains supplementary material, which is available to authorized users.

## Background

Soybean (*Glycine max* L. Merr.) is a legume species that is widely planted in the worldwide. This legume is an excellent source of vegetable protein, that is, vegetable oil, and it contains many special nutrients, such as isoflavone, phytosterol, and saponin [1]. However, in some developing countries, with the rapid industrialization and urbanization, as well as large population base, the arable land resources have become increasingly depleted, and the gap between soybean supply and demand continuously increases [2]. Intercropping systems are beneficial for biodiversity conservation and increase crop yield in many countries [3–5]. Examples of intercropping patterns include wheat–maize, maize–soybean, sunflower–soybean, and sorghum–soybean. Among these examples, maize–soybean intercropping is a major planting pattern in the worldwide; it remarkably enhances soybean production while maintaining the maize yield [6, 7]. However, during the symbiotic period of maize–soybean intercropping system, tall maize plants directly change the light environment, especially the quality and intensity of light, of the soybean. Most previous studies on the photosynthetic response of crops to shading mainly used artificial shading or single light, which does not change the spectrum components.

Light plays a major role in photosynthetic capacity; it not only provides the driving force for photosynthesis but also affects the leaf structure and function [8]. Soybean, which is a short crop type, is highly sensitive to shading; under an intercropping system, soybean changes morphologically and physiologically to adapt to the alteration of its light environment by the neighboring tall crops [6]. Relevant studies on shade environment revealed that soybean plants respond to changes in light radiation through sufficient physiological flexibility [9, 10]. Leaves are the major organ for photosynthesis of soybean plant, and their development substantially affects soybean growth, resistance, yield, and quality [11]. Soybean leaves in shade condition are indicated by a decline in the photosynthetic rate and leaf area [12]. Generally, leaves under the shaded condition are smaller and thinner than those of under the normal light (CK), the palisade tissue cells are undeveloped with relaxed arrangement, the chloroplast is smaller than those of under CK treatment, and the grana lamellae are tightly stacked. Previous studies indicated that thinner leaves have more chance to intercept light than thicker one, but this structure is a disadvantage for the fixation and transport of CO_2_ [13–15].

Protein is an important material basis and directly involved in life activities and physiological functions. Therefore, the changes in life activities under different environmental conditions can be interpreted through the study of protein functions and expression levels. Proteomics is linked to plant phenotypes and genotypes and plays a vital role in the study of plant resistance [16, 17]. The application of proteomics in the stress resistance of soybean plants mainly includes biotic and abiotic stresses. Previous studies used proteomics techniques to study soybean flooding [18], salt [19], drought [20], and shading stresses on sorghum [14] and found that some key differential protein, thereby revealing the protein network pathway of stress and constructing the corresponding regulatory model. However, relevant studies on the photosynthesis in soybean leaves under the shading treatment with maize are lacking.

Proteomics technologies are effective tools for the analysis of protein function. Two-dimensional electrophoresis (2-DE) can separate different proteins in high resolution due to the differences in isoelectric point and molecular weight. However, 2DE has some disadvantages rendering it unsuitable for the detection of low-abundance proteins [11, 21]. With high-throughput, high-sensitivity, and high-resolution biological mass spectrometry technologies, proteomics technology has achieved rapid development. Isobaric tags for relative and absolute quantification (iTRAQ) is a mature quantitative proteomics technique by Applied Biosystems Inc. (USA), and it has become prevalent in developmental proteomics because it can identify and quantify proteins from multiple samples with high coverage [22–24]. In this study, we attempted to elucidate the mechanism underlying the response to shading of soybean leaves in intercropping mode by analyzing photosynthetic rate, leaf structure, physiological and differential proteome changes in soybean.

## Methods

### Plant materials

A soybean plant cultivar, that is, Nandou12, was grown in two treatments, namely, CK (Additional file 1a) and shading (maize–soybean relay intercropping, Additional file 1b). The seeds of ‘Nandou 12’ were provided by the Nanchong Institute of Agricultural Sciences, Sichuan Province, China. Maize cultivar ‘Chuandan 418’, from the Maize research Institute of Sichuan Agricultural University, Sichuan Province, China. Experiment was conducted in a greenhouse in the College of Agronomy, Sichuan Agricultural University, Chengdu, Sichuan province, China (30°71′20″N, 103°86′87″E). Soybean seeds were sown in rectangular plastic basins (length: 43.5 cm, width: 20 cm and height: 14 cm) filled with humidified nutrition soil. In the greenhouse, the day/night temperature and air relative humidity were 25 °C/20 °C and 60%, respectively. In this experiment, soybean plants were subjected to two irradiance levels, that is, 100% (unshaded) and 10.9% (shade), for 15 days. Shading treatment was conducted intercropping with maize. Five pots per each treatment and eight plants with V3 stage of soybean per pot.

The spectral irradiance of soybeans canopy and photosynthetically active radiation (PAR, wavelength in the range of 350–1000 nm) under normal light and shade treatments were measured with a spectrograph (AvaSpec-2048) and a quantum sensor (LI-1400, LI-COR, USA). All plants were kept well irrigated and protected from bacterial pathogens and weed competition. The spectral radiance values at different wavelengths under shading treatment were lower than those of under CK treatment (Additional file 1c). The PAR under shading treatment was significantly lower than that in CK treatment (Additional file 1d). In particular, spectral radiance from blue to far-red light (Fr) exhibited decreasing trend. The ratio of red light (R, 655–665 nm) to Fr (725–735 nm) (R/Fr ratio) varied considerably during plant growth and development (Additional file 1c). Therefore, a large difference in the R/Fr ratio of the soybean canopy was observed between CK (R/Fr ratio = 1.31) and shading treatment (R/Fr ratio = 0.37). Three independent biological replicates with soybean leaves were used to conduct the proteomic analysis and leaf samples were frozen in liquid nitrogen and stored at − 80 °C.

### Measurement of pigment content and photosynthetic rate

Chlorophyll and carotenoids were extracted with 80% acetone from the two leaf discs of a fresh soybean plant sample at the V3 stage by using punch hole method. The extract was measured spectrophotometrically (DU-730, Beck Man Coulter, USA) at 646, 663, and 470 nm. The photosynthetic rate (*P*_*n*_), intercellular CO_2_ concentration (*C*_*i*_) and stomatal conductance (*G*_*s*_) were determined from 09:30 to 11:00 on a sunny day in a leaf chamber with a CO_2_ concentration of 400 μmol mol^− 1^ and a relative humidity of 60% under an irradiance of 600 mmol m^− 2^ s^− 1^ by using a photosynthetic system (LI-6400, LI-COR, USA). Nine leaves of soybean plants were measured for each planting density.

### Measurement of morphology, stomatal density and leaf structure

At V3 stage of soybean growth, plant height and hypocotyl length of five soybean seedlings were measured. Stomatal density was measured by nail polish methods [25]. Nail polish was applied to the soybean leaf to obtain a replica of the leaf surface. The replica was observed using light microscope (Nikon Eclipse 50i, Japan). The number of stomata was counted in 15 fields of view using five marked leaves from each treatment.

Leaf segments (5 mm × 10 mm) without veins were fixed at 4 °C in a FAA solution (38% formaldehyde/glacial acetic acid/70% alcohol, 5:5:90, *v*/v). Leaf segments of soybean were used to prepare paraffin sections. Light microscopy analysis (Nikon Eclipse 50i, Japan) was performed using a 10 μm thick transverse section of the leaf stained with fast green and counterstained with safranin. The thicknesses of the leaf, palisade, and spongy mesophyll tissues were obtained using ImageJ 1.42q.

### Measurement of the chloroplast ultrastructure

Soybean leaf was prefixed with a mixed solution of 3% glutaraldehyde, and then it was postfixed in 1% osmium tetroxide. Leaf was dehydrated using a series of acetone, infiltrated in Epox 812, and then embedded. Semithin sections were stained with methylene blue. Ultrathin sections were cut with a diamond knife and stained with uranyl acetate and lead citrate. Sections were examined under a transmission electron microscope (TEM; HITACHI, H-600IV, Japan). Chloroplast cover index (proportion of cell area occupied by chloroplasts) was prepared using Image J 1.42q. For each thin section, mesophyll cells were examined to determine the chloroplast and starch grain sizes.

### Protein extraction

Total proteins were extracted from the Nandou12 leaf tissue. Sample was first grinded by liquid nitrogen, and then the cell powder was sonicated thrice on ice by using an ultrasonic processor (Scientz) in a lysis buffer (2 mM EDTA, 8 M urea, 1% protease inhibitor cocktail, and 10 m MDTT). The remaining debris was removed by centrifugation at 20,000 g and 4 °C for 10 min. Finally, protein was precipitated with cold 15% TCA for 2 h at − 20 °C. After centrifugation at 4 °C for 10 min, the supernatant was discarded. The remaining precipitate was washed thrice with cold acetone. The protein was redissolved in a buffer (100 mM TEAB, 8 M urea, and pH of 8.0), and its concentration was determined with a 2D Quant kit according to the manufacturer’s instructions. For digestion, the protein solution was reduced with 10 mM DTT for 1 h at 37 °C and alkylated with 20 mM IAA for 45 min in the dark at room temperature. For trypsin digestion, the protein sample was diluted by adding 100 mM TEAB to a urea concentration of < 2 M. Finally, trypsin was added at the trypsin-to-protein mass ratio of 1:50 for the first digestion overnight and the trypsin-to-protein mass ratio of 1:100 for the second digestion for 4 h. Approximately 100 μg protein for each sample was digested with trypsin for the succeeding experiments.

### iTRAQ labeling and HPLC fractionation

After trypsin digestion, the peptide was desalted using Strata X C18 SPE column (Phenomenex) and then vacuum dried. The peptide was reconstituted in 0.5 M TEAB and processed in a 4-plex iTRAQ kit according to the manufacturer’s protocol. Briefly, a unit of the iTRAQ reagent (defined as the amount of reagent required to label 100 μg protein) was thawed and reconstituted in 24 μl of ACN. Then, the resulting peptide mixtures were incubated for 2 h at room temperature, and then it was pooled, desalted, and dried by vacuum centrifugation. Afterward, the sample was fractionated via high-pH reverse-phase HPLC by using Agilent 300 Extend C18 column (5 μm particle size, 4.6 mm ID, and 250 mm length). Briefly, peptides were first separated with a gradient of 2 to 60% acetonitrile in 10 mM (NH_4_)HCO_3_ (pH of 10) for over 80 min into 80 fractions. Then, peptides were combined into 18 fractions and dried via vacuum centrifugation.

### Quantitative proteomic analysis via LC–tandem mass spectrometry (MS/MS)

Peptides were dissolved in 0.1% FA and directly loaded onto a reverse-phase analytical column (Acclaim PepMap RSLC, Thermo Scientific). At a constant flow rate of 350 nL/min on an EASY-nLC 1000 UPLC system, the gradient comprised an increase in solvent B from 6 to 25% (0.1% FA in 98% ACN) in over 42 min from 25 to 40% in 12 min, reached to 80% in 4 min, and then remained at 80% for the last 4 min. The resulting peptides were analyzed using an Orbitrap Fusion™ Tribrid™ mass spectrometer (Thermo Fisher Scientific). The peptides were subjected to a NSI source, followed by MS/MS in an Orbitrap Fusion™ Tribrid™ (Thermo Fisher Scientific) coupled online to UPLC. Intact peptides were detected in the Orbitrap at a resolution of 60,000. Peptides were selected for MS/MS by using 38 as the NCE setting, and ion fragments were detected in the Orbitrap at a resolution of 15,000. A top speed data-dependent procedure alternating between one MS scan and the most intense MS/MS scan was applied for the precursor ions above the threshold intensity greater than that of 1E4 in the MS survey scan with 30 s dynamic exclusion. The applied electrospray voltage was 2.0 kV. Automatic gain control was used to prevent Orbitrap overfilling. 1E5 ions were accumulated for MS/MS spectra generation. For MS scans, the m/z scan range was 400–1600, and the fixed first mass was set at 100 m/z.

### Proteomic analysis of soybean leaf

The resulting MS/MS data were processed using MaxQuant with integrated Andromeda search engine (v.1.5.2.8). Tandem mass spectra were searched against the Uniprot *Glycine max (Linn.) Merr* database concatenated with a reverse decoy database. Trypsin/P was specified as the cleavage enzyme allowing a maximum of two missing cleavages. The mass error was set at 10 ppm for precursor ions and 0.02 Da for fragment ions. Carbamidomethylation on Cys was specified as the fixed modification, and oxidation on Met and acetylation on protein N-terminal were specified as variable modifications. False discovery rate thresholds for proteins, peptides, and modification sites were specified at 1%. The minimum peptide length was set at 7. iTRAQ-8plex was selected for the quantification method. All the other parameters in MaxQuant were set to default values.

### Real-time quantitative PCR analysis

The RNA was isolated with miRNeasy mini kit (QIAGEN, 217004, Germany). Real-time quantitative PCR (qRT-PCR) assay [25]. The beta-tubulin gene was used as the reference control in the study. Real-time PCR was measured with a CFX96 system (Bio-Rad, USA). The primers are listed in Additional file 2.

### Statistical analyses

SPSS version 19.0 was utilized to compare data through one-way ANOVA and test the differences between shading and CK treatments. ANOVA was used in a Tukey–Kramer comparison for significant differences at the 5% level in all parameters. For all statistical analyses, at least three biological replicates were used for treatment and control. Microsoft Excel 2016 was used for data calculation. Origin Pro 9.1 and Microsoft Excel were used to draw the figures.

## Results

### Changes in the chlorophyll content and photosynthetic rate

**Fig. 1:**
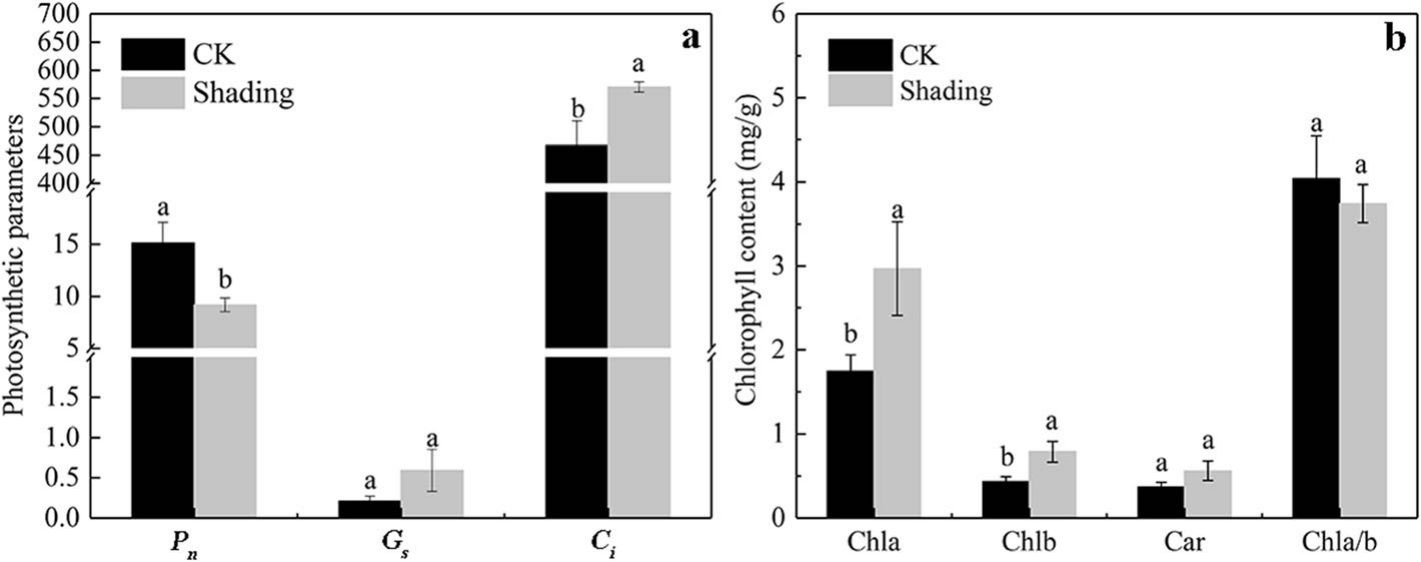
Photosynthetic rate (**a**) and pigment contents (**b**) of soybean leaves under shading and CK treatments. *P*_*n*_: photosynthetic rate, *G*_*s*_: stomatal conductance, *C*_*i*_: intercellular CO_2_ concentration, Chl a: chlorophyll a content, Chl b: chlorophyll b content, Car: carotene content, Chl a/b: the ratio of Chl a to Chl b. Significant differences between shading and CK treatments are indicated by different small letters (*P* < 0.05), respectively

Plants under shading treatment exhibited low *P*_*n*_. Compared with CK treatment, *P*_*n*_ value of leaves under shading treatment significantly decreased by 39.3%, but their *C*_*i*_ value significantly increased by 22.0%. *G*_*s*_ value showed insignificant difference (Fig. 1-a). The major photosynthetic pigments found in soybean leaves were chlorophyll a (Chl a), chlorophyll b (Chl b), and carotene (Car). The Chl a, Chl b and Car in the leaves under shading treatment were higher than those of leaves under CK treatment by approximately 70.4, 83.2 and 52.6%, respectively, but Chl a/b was lower than that of control by 7.3% (Fig. 1-b).

### Changes in leaf stomatal density

**Fig. 2:**
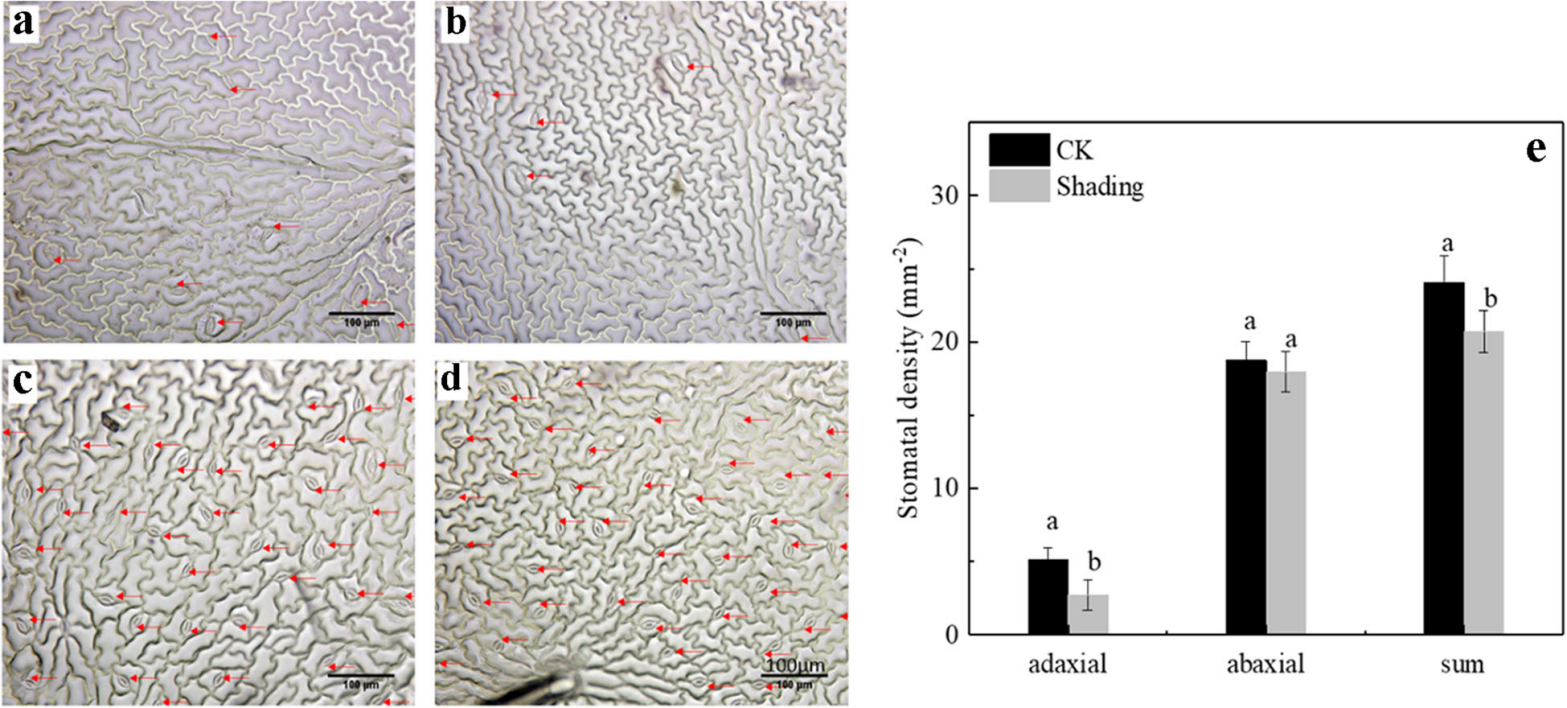
Stomatal density of soybean leaves under shading and CK treatments. Stomatal density on the adaxial side of leaves under CK (**a**) and shading (**b**) treatments. Stomatal density on the abaxial side of leaves under CK (**c**) and shading (**d**) treatments. Significant differences between shading and CK treatments are indicated by different small letters (*P* < 0.05), respectively

In this study, the stomatal densities on the adaxial and abaxial sides of soybean leaves under shading treatment were 2.74 and 17.97 mm^− 2^, respectively (Fig. 2-e). The adaxial and total stomatal densities under shading were significantly lower than those of under CK by 46.5 and 14.0%, respectively. However, the abaxial stomatal densities of CK and shading treatments showed insignificant difference (Fig. 2).

### Changes in morphology and leaf anatomical structure

**Fig. 3:**
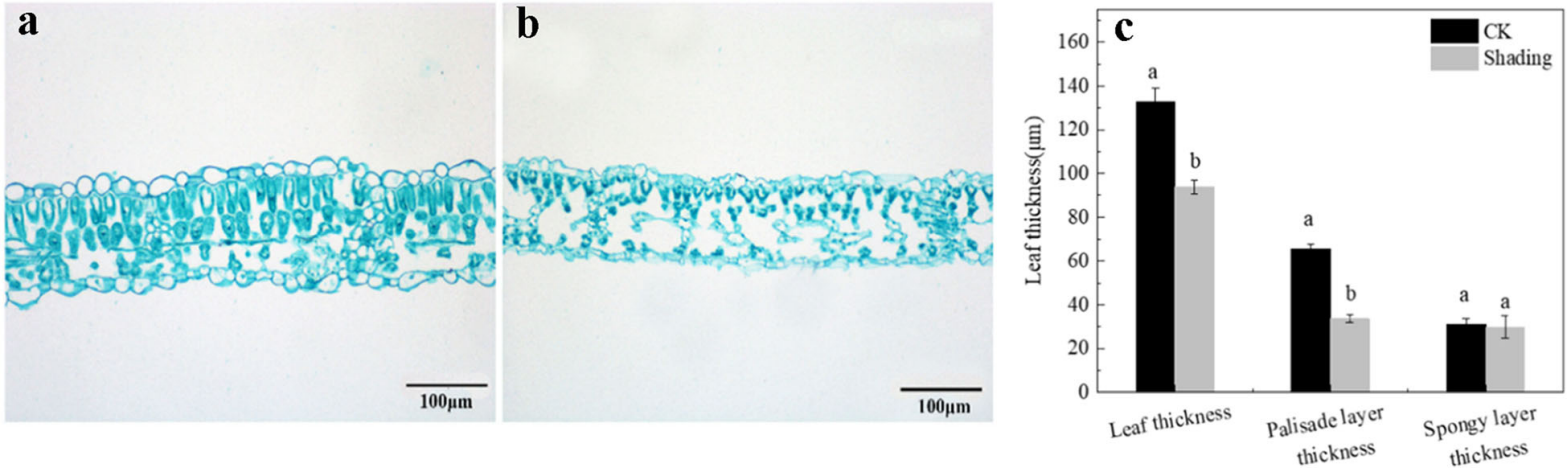
Anatomical structure of soybean leaves under CK and shading treatments. Significant differences between shading and CK treatments are indicated by different small letters (*P* < 0.05), respectively

We also analyzed the soybean plant, hypocotyl length and leaf structure, as shown in Additional file 3 and Fig. 3. Plant height and hypocotyl length of the soybean under shading significantly increased by 63.8 and 59.8%, compared with CK. The soybean leaf type was bifacial, and the mesophyll tissue differentiated into palisade and sponge tissues. The upper and lower epidermal cells, the palisade and spongy cells of the leaves of plants under shading were smaller and arranged loosely with larger intercellular gap than those of under CK treatment (Fig. 3-a, b). Shading decreased the leaf, palisade tissue, and sponge tissue thicknesses of the leaf compared with under CK treatment. The leaf and palisade tissue thicknesses under shading treatment decreased significantly by 29.2 and 48.9%, respectively. The difference in the sponge thicknesses under shading and CK treatments was insignificant. Therefore, the changes in the anatomical structure indicated that the soybean plant grown under shading treatment developed typical features of shade plants (Fig. 3).

### Changes in chloroplast ultrastructure

**Fig. 4:**
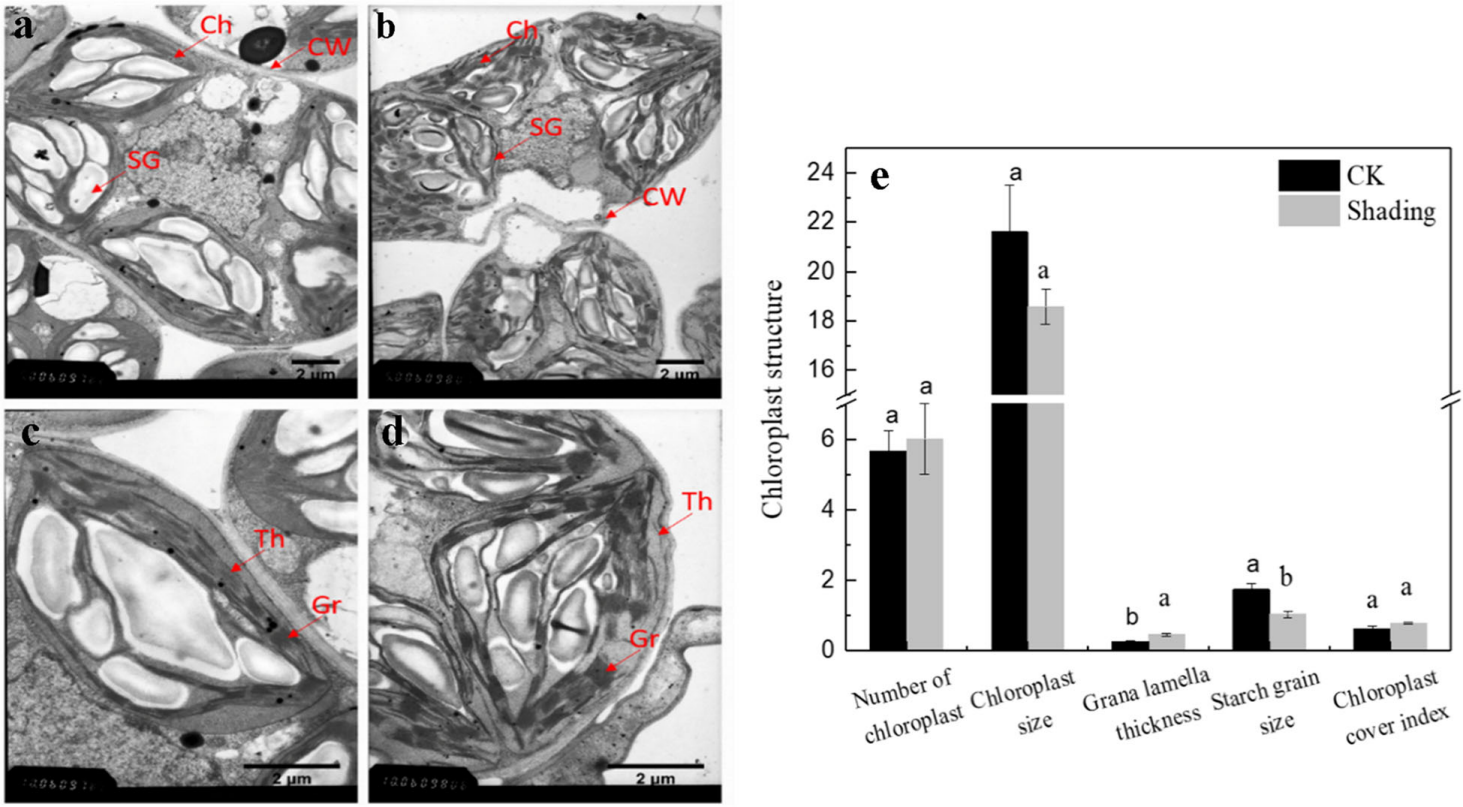
Chloroplast ultrastructure of soybean leaves under shading and CK treatments. **a**, **c** CK treatment and **b**, **d** shading treatment. Ch: chloroplast, CW: cell wall, Gr: grana, and Th: thylakoid. Significant differences between shading and CK treatments are indicated by different small letters (*P* < 0.05), respectively

TEM analysis revealed that the leaves of plants under shading treatment exhibited the typical chloroplast ultrastructure consisting of grana and thylakoids (Fig. 4). In plants under shading treatment, the shape, size, and number of chloroplasts were significantly affected (Fig. 4-b). The chloroplast structure of soybean leaves under shading treatment was essentially intact, but some chloroplasts also appeared irregularly elliptic, with the middle part raised to the cell wall. The cell, chloroplast, and starch grain sizes decreased under shading treatment, but the number of chloroplasts and the thickness of grana layer and the chloroplast cover index, increased (Fig. 4). The thickness of the grana lamella under shading treatment was significantly higher than that of the control by 72.3%, while starch grain size was significantly lower than that of the control by 40.1% (Fig. 4-e).

### Proteomic analysis of soybean leaf

#### GO classification

**Fig. 5:**
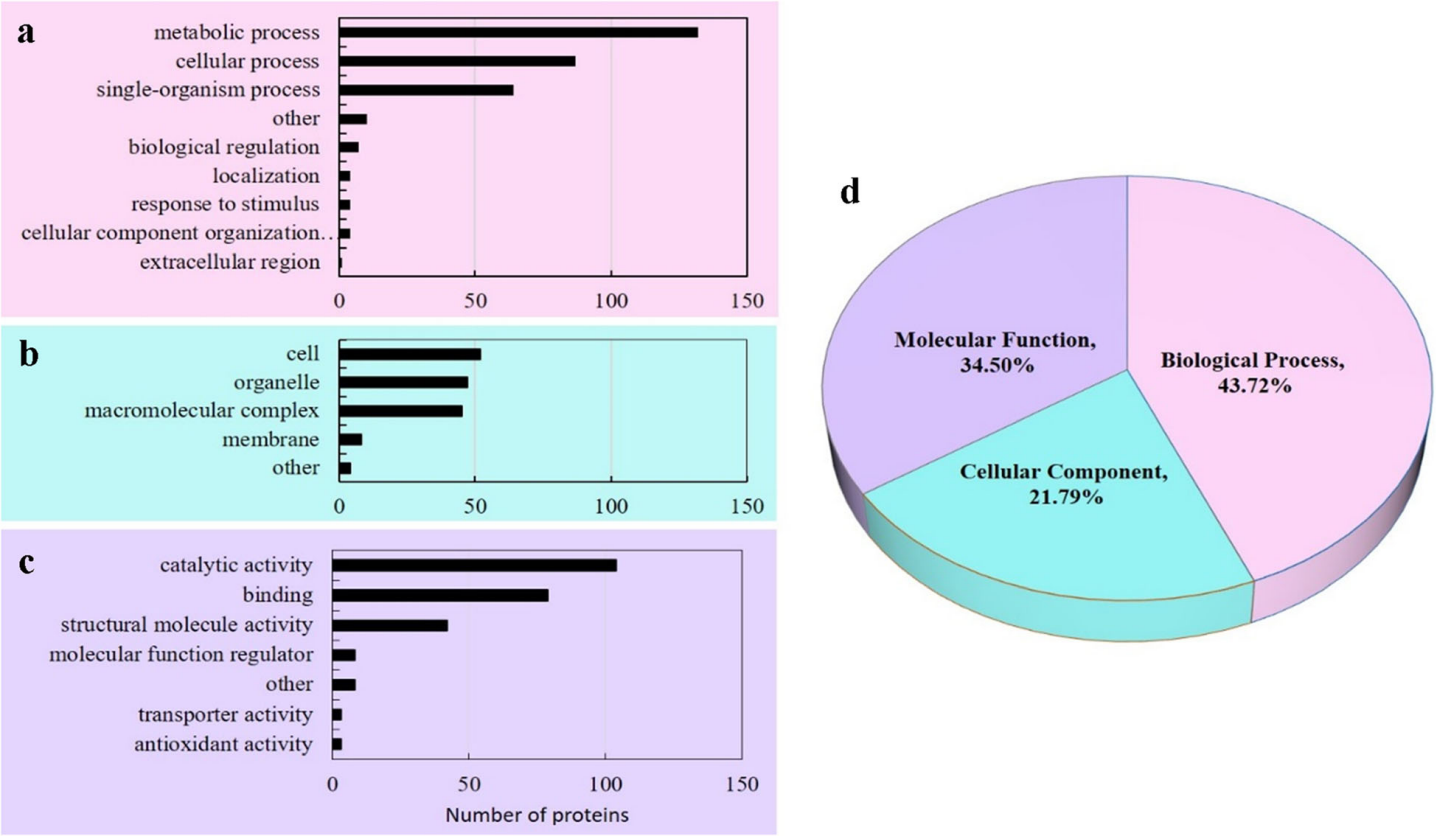
GO annotation of differentially abundant proteins in soybean leaves under shading treatment. GO are classified into three domains, namely, (**a**) biological process, (**b**) molecular function, and (**c**) cellular component

The fold-change (FC) cut-off was set when proteins with quantitative ratios above 1.3 or below 0.77 are deemed significant. A total of 248 differentially expressed proteins from the leaves under shading and CK treatments were detected through iTRAQ labeling in three biological repeats, reproducibility analysis of three repeated trials by Pearson correlation coefficient (Additional file 4). Among these proteins, 138 proteins were upregulated, and 110 proteins were downregulated (*P* < 0.05, FC > 1.3, FC < 0.77) (Additional file 5). According to GO annotation information of identified proteins, the differentially expressed proteins were classified into three large categories (biological process: 43.72%, cellular component: 21.79%, and molecular function: 34.50%) and 20 small groups (Fig. 5-d). The top three categories for each functional group were as follows: metabolic, cellular, and single-organism processes for biological process (Fig. 5-a); cell, organelle, and macromolecular complex for the cellular component (Fig. 5-b); and catalytic activity, binding, and structural molecule activity for the molecular function (Fig. 5-c).

#### Subcellular location classification

**Fig. 6:**
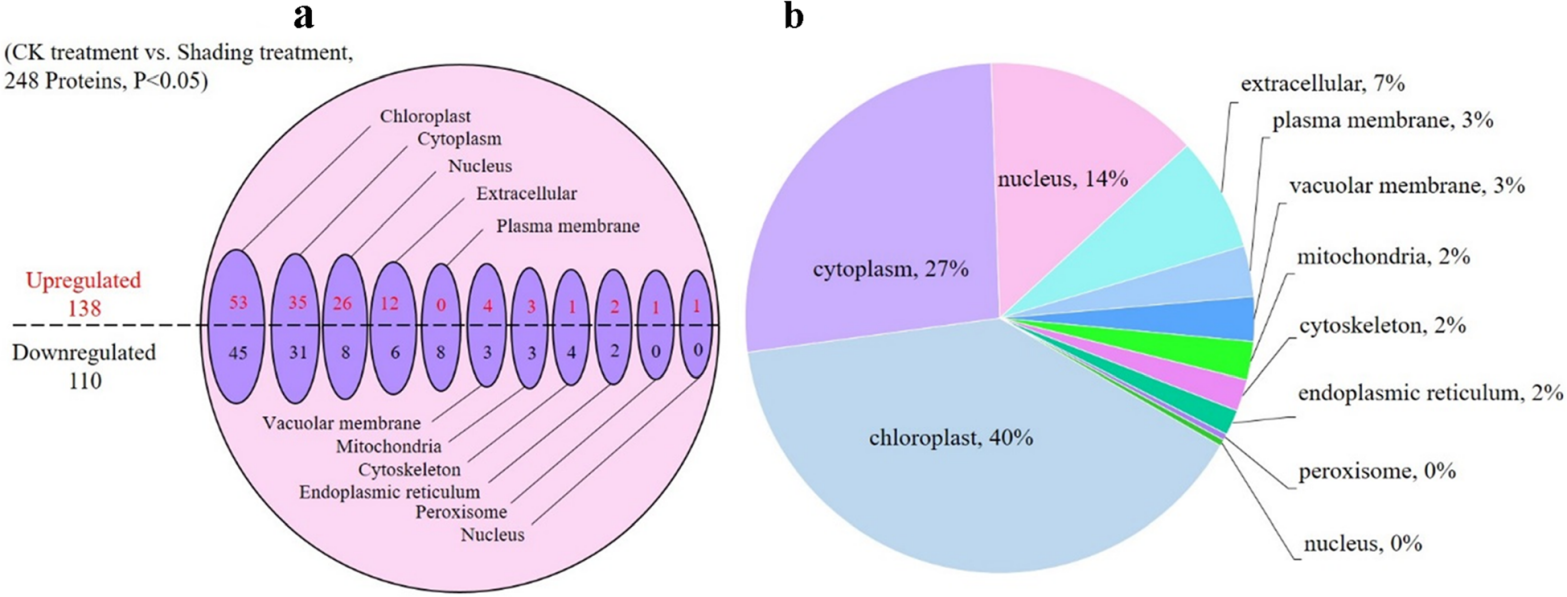
Subcellular locations of proteins in soybean leaves under shading

To improve our understanding on the function of differentially expressed proteins and determine the effect of intercropping on the physiological and biochemical pathways of soybean leaves, we analyzed these proteins in soybean leaves via subcellular localization. Figure 6 shows that 248 differential proteins were localized as follows: 98 (40%) were located in the chloroplast, 66 (7%) in the cytosol, 34 (14%) in the nucleus, 18 (7%) in the extracellular region, 8 (3%) in the plasma membrane, 7 (3%) in the vacuolar membrane, 6 (2%) in the mitochondria, 5 (2%) in the cytoskeleton, and 4 (2%) in the endoplasmic reticulum. Hence, differential proteins are mainly located in the chloroplast, cytoplasm, and nucleus; some proteins are situated in the cell membrane, vacuole, mitochondria, endoplasmic reticulum, and cytoskeletal structures.

#### Changes in the protein expression in soybean leaves

To determine the biological functions and interactions of the differentially expressed proteins, we further investigated the identified differential proteins by using the Kyoto Encyclopedia of Genes and Genomes (KEGG) database. A total of 13 differentially expressed proteins were mapped into KEGG pathways. These pathways included the porphyrin and chlorophyll metabolism, photosynthesis-antenna proteins, photosynthesis, carbon fixation in photosynthetic organisms (Table 1). These proteins included four proteins in porphyrin and chlorophyll, three photosynthesis-antenna proteins, two in carbon fixation in photosynthetic organisms and one in photosynthesis were significantly upregulated under shading treatment. Two proteins involved in photosynthesis and one in photosynthesis-antenna protein were significantly downregulated under shading treatment compared with CK (Table 1).

**Table 1:** Annotation of the major differentially expressed proteins in shading

Protein accession	Protein description	Fold change	*P* value	Regulated type	Subcellular localization
Porphyrin and chlorophyll metabolism					
A0A0R4J3L3	Protochlorophyllide reductase (por)	2.115	0.001	Up	chloroplast
Q9XE94	Geranylgeranyl hydrogenase (Ggh)	1.355	0.029	Up	chloroplast
A0A0R4J4S6	Protochlorophyllide reductase	1.444	0.002	Up	chloroplast
A0A0R0H273	Tetrapyrrole-binding protein (TBP)	1.769	0.001	Up	chloroplast
Photosynthesis-antenna proteins					
A0A0R4J5I3	light-harvesting complex II chlorophyll a/b binding protein 1 (LHCB1)	3.765	0.016	Up	chloroplast
Q93YG3	light-harvesting complex II chlorophyll a/b binding protein 2 (LHCB2)	1.413	0.026	Up	chloroplast
I1KR46	light-harvesting complex II chlorophyll a/b binding protein 6 (LHCB6)	1.319	0.047	Up	chloroplast
I1MZ32	light-harvesting complex II chlorophyll a/b binding protein 4 ((LHCB4)	0.744	0.028	Down	chloroplast
Photosynthesis					
I1M3X9	Protein THYLAKOID FORMATION1 (PTF)	1.445	0.009	Up	chloroplast
I1LLA7	NADPH:quinone oxidoreductase (NQO)	0.623	0.033	Down	chloroplast
A0A0R0IP95	Ferredoxin (Fd)	0.706	0.001	Down	chloroplast
Carbon fixation in photosynthetic organisms					
I1J4J6	Malic enzyme	1.314	0	Up	chloroplast
Q9FUJ5	Ribulose bisphosphate carboxylase small chain (rbcS)	1.407	0.007	Up	chloroplast

#### Gene expression

**Fig. 7:**
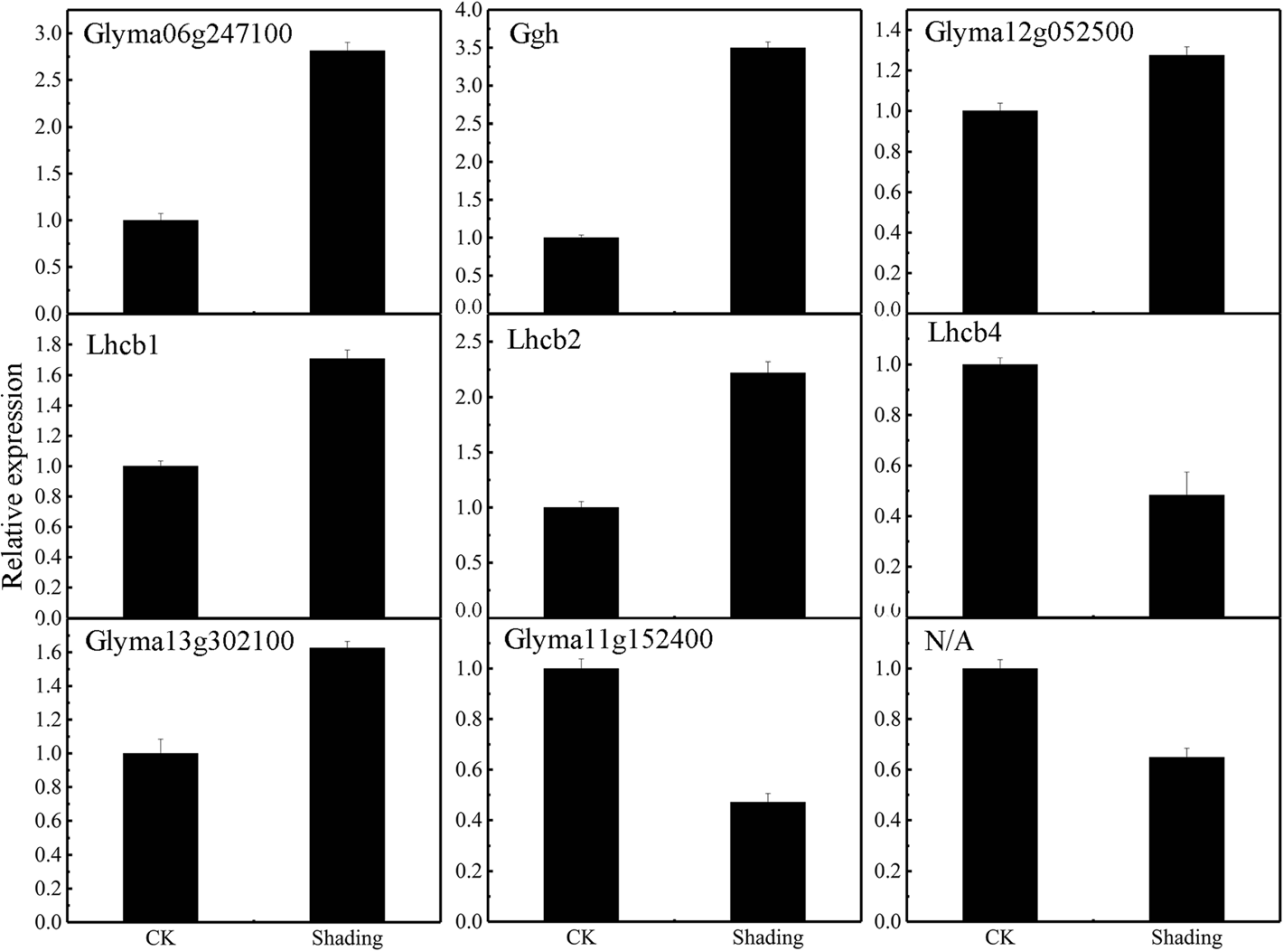
Expression analysis of nine soybean leaf genes in CK and shading treatments using Real-time RT-PCR. Glyma06g247100: Protochlorophyllide reductase (por); Ggh: Geranylgeranyl hydrogenase (Ggh); Glyma12g052500: Tetrapyrrole-binding protein (TBP); Lhcb1: light-harvesting complex II chlorophyll a/b binding protein 1 (LHCB1); Lhcb2: light-harvesting complex II chlorophyll a/b binding protein 2 (LHCB2); Lhcb4: light-harvesting complex II chlorophyll a/b binding protein 4 ((LHCB4); Glyma13g302100: Protein THYLAKOID FORMATION1 (PTF); Glyma11g152400: NADPH:quinone oxidoreductase (NQO); *N/A*: Ferredoxin (Fd)

To validate the identified differentially expression proteins, nine gene products were subjected to qRT-PCR analysis (Fig. 7). The gene expression levels of Glyma06g247100, Ggh, Glymal2g052500, Lhcb1, Lhcb2, Glymal3g302100 under the shading treatment were significantly higher than those under the CK. But, the gene expression levels of Lhcb4, Glymallg152400 and N/A under the shading were significantly lower than those under the CK. These results were consistent with the profiles of differentially expressed proteins insoybean leaves identified through i TRAQ analysis.

## Discussion

### Photosynthesis of soybean leaves in response to shading

Plants have evolved to multiple phenotypic plasticity adaption to different light environments [26]. The growth of short plants in intercropping can be strongly affected by light competition from high plants. In the present study, maize and soybean intercropping resulted in substantial shading to soybean, which was reflected by a remarkable decline in spectral irradiance (Additional file 1c) and PAR (Additional file 1d). In our study, the soybean plant height and hypocotyl length were increased under shading (Additional file 3). This indicated that the soybean plant appeared most striking shade avoidance syndrome. Shade avoidance responses enhance light capture at high plant densities [27].

Photosynthesis is the primary assimilation process in plants [28]. Photosynthetic rate indicates the photosynthetic ability of plant organs [29]. Our results showed that under these conditions, leaf function is affected by the decrease in PAR and spectral irradiance, which also decreases photosynthesis. The *C*_*i*_ in leaves under shading significantly increased by 22.0% compared with those under CK treatment. The CO_2_ concentration may be the main restricting factor in decreasing the photosynthetic rate in the leaves of the soybean plants under CK and shading treatments. At the same time, the *G*_*s*_ in soybean plants changed slightly under shading treatment (Fig. 1-a). Chlorophyll is the main pigment for plants in absorbing and utilizing light energy. The changes in chlorophyll content indicate the advantages and disadvantages of the initial photosynthetic reaction. In particular, Chl a directly influences the photosynthetic activity and increases the Chl and *P*_*n*_ levels [30]. The results of our study showed that the soybean leaves under shading treatment exhibited increased Chl and Car contents per unit mass (Fig. 1-b). Plant leaf contained more chlorophyll content for a high efficient capture of light. The reduction of the Chl a/b ratio in shade were most likely due to changes in the organization of both light-intercepting and electron transport components [31].

### Leaf structure of soybean leaves in response to shading

Leaf structure provides a structural framework for the diffusion of gases and optimization of photosynthetic function [32]. Theoretically, high stomatal density and thick leaves, as well as a rapid metabolite transfer among mesophyll cells, tend to favor high photosynthetic rate. Therefore, under shading treatment, reduced stomatal density, palisade tissue thickness, spongy thickness, and leaf thickness may lead to decreased photosynthetic capacity (Fig. 1). The density and size of stomata mainly affect the maximum stomatal conductance potential [33]. In a previous study, the stomatal density increases, but the stomatal size decreases, this result is due to the quick response of small stomata to rapidly changing environments [34]. In the present study, under shading, the stomatal density significantly decreased, but *C*_*i*_ increased, and *G*_*s*_ was similar to that under CK treatment. This result may be due to decreased photosynthetic activity of mesophyll cells.

Photosynthesis occurs in the chloroplasts of plant cells, and it effectively transforms sun energy into chemical energy [35]. The most prominent structure in the chloroplast is the inner membrane branch system of the thylakoid, and the chlorophyll and carotenoids that are involved in photosystems are located in grana, which are made up of thylakoids [36]. In this study, the number of chloroplasts and the thickness of grana layer and the chloroplast cover index of soybean leaves under shading were increased (Fig. 4). The increased number of grana layers in chloroplasts enhance the light absorption for the transfer and convert of chloroplast functions, which were beneficial to the formation and accumulation of photosynthetic products [37]. However, in the present study, the chloroplasts in soybean leaves of plants treated under shading contained lower starch grains, and the size of the starch grain was considerably lower than those of the control. This indicated that in order to adapt the shade, soybean plant enhance the light absorption and the transfer of PSII, but these light energy is not completely converted into photosynthetic products.

### Protein expression in soybean leaves under shading

Leaves of the soybean plant are the primary sites of photosynthesis. In this study, proteomic analysis of the leaves of the soybean identified 248 differentially proteins in shading by iTRAQ (Additional file 5). Four proteins were involved in porphyrin and chlorophyll metabolism were upregulated (Table.1). Chl and car are the main pigments that trap energy in the form of light. Altered Chl metabolism is one of the most important factors leading to the chlorophyll content increased. Simultaneously, we found that four proteins (por, Ggh and TBP) involved in the Chl metabolism exhibited upregulated expression levels in shading (Table.1). The proteins involved in the Chl biosynthesis pathway were upregulated expression lead to increased Chl content in shading.

Photosynthesis takes place in the chloroplast [38], changes of the chloroplast structure and altered Chl biosynthesis may affect the photosynthetic capacity of soybean plants. The differentially proteins identified in this study were significantly enriched in the photosynthesis pathway (Table 1). The expression of light-harvesting complex II chlorophyll a/b binding proteins (LHCII) of soybean leaves in shading were upregulated. In higher plants, the light-harvesting pigment protein complex (LHC) is a pigment-protein complex that captures light energy and rapidly transfers energy to the reaction center to cause photochemical reactions [39]. In addition to the absorption and transmission of light energy in the thylakoid membrane, they maintain the structure of the thylakoid membrane, regulate the distribution of excitation energy between the two optical systems, photoprotection, and adaptation to various environments [40]. However, expression of NADPH:quinone oxidoreductase (NQO) and Ferredoxin (Fd) in the chloroplasts of soybean leaves under shading were downregulated. NQO is involved in a variety of energy reactions, including cellular respiration, cyclic electron transport around PSI, and CO_2_ uptake. In the photoreaction process, Fd acts as an electron transporter of the photoreaction terminal, mediates electron transfer between PSI and CO_2_ fixation, and electron reflow in photosynthetic cycle phosphorylation [41]. This results indicated that shade condition enhance the light capture efficiency of PS II in soybean leaves, but decreased the capacity from PSII transmitted to PSI.

Therefore, the light-harvesting complex was the main regulatory site of leaf photosynthesis under shading treatment. This result may be advantageous to the PS II stability and activity [42]. Shading were also downregulated the expression of proteins in PSI, thereby possibly limiting the normal function. The Calvin cycle involves numerous enzymes, including Rubisco that is commonly regarded as a key enzyme in photosynthesis. Our data indicated that the upregulation of the ribulose bisphosphate carboxylase small chain (rbcS) was observed under shading treatment (Table 1). Therefore, rubisco may not be a rate-limiting photosynthetic step under shading conditions.Our study indicated that differentially expressed genes and proteins be mapped to the ‘porphyrin and chlorophyll metabolism,’ ‘photosynthesis-antenna proteins,’ and ‘photosynthesis’ Pathways. In this pathway, nine genes were involved in photosynthesis in the soybean leaves were selected for real-time PCR validation to determine if gene expression data would confirm the changes in protein abundance. The expression of nine genes (Glyma06g247100, Ggh, Glyma12g052500, Lhca1, Lhca2, Lhca4, Glyma13g302100, Glyma11g152400 and N/A) were consistent with the proteomic data (Fig. 7, Table 1).

## Conclusion

Our results demonstrated that the decline of photosynthesis in soybean under shade condition involves the regulation of photosynthetic pigment content, leaf structure, chloroplast ultrastructure and levels of proteins related to photosynthesis. Most proteins involved in porphyrin and chlorophyll metabolism, photosynthesis-antenna proteins, carbon fixation in photosynthetic organisms were upregulated. By contrast, proteins involved in photosynthesis were downregulated. These results demonstrated that shade condition increased the light capture efficiency of PSII in soybean leaves but decreased the capacity from PSII transmitted to PSI. This maybe the major reason that the photosynthetic capacity was decreased in shading.

## Additional files

Additional file 1: Spectral irradiance (c) and PAR (d) of soybean canopy. a: normal light, b: maize–soybean relay strip intercropping. Significant differences between CK and shading treatments are indicated by different small letters (*P* < 0.05), respectively. (TIF 2260 kb)

Additional file 2: Real-time PCR primers. (PDF 309 kb)

Additional file 3: Plant height (a) and hypocotyl length (b) of soybean plant. Significant differences between CK and shading treatments are indicated by different small letters (*P* < 0.05), respectively. (TIF 344 kb)

Additional file 4: MS identified information. (XLSX 2750 kb)

Additional file 5: Differentially expressed statistics. (XLSX 62 kb)

## Abbreviations

2-DE: Two-dimensionalgel electrophoresis
Ch: Chloroplast
*C*_*i*_: Intercellular CO_2_ concentration
CW: Cell wall
Fd: Ferredoxin
Ggh: Geranylgeranyl hydrogenase
GO: Gene Ontology
Gr: Grana
*G*_*S*_: Stomatal conductance
iTRAQ: Isobaric tags for relative and absolute quantification
LHCB1: Light-harvesting complex II chlorophyll a/b binding protein 1
LHCB2: Light-harvesting complex II chlorophyll a/b binding protein 2
LHCB4: Light-harvesting complex II chlorophyll a/b binding protein 4
LHCB6: Light-harvesting complex II chlorophyll a/b binding protein 6
LHCII: Light-harvesting complex II chlorophyll a/b binding proteins
NQO: NADPH:quinone oxidoreductase
*P*_*n*_: Photosynthetic rate
por: Protochlorophyllide reductase
PTF: Protein THYLAKOID FORMATION1
rbcS: Ribulose bisphosphate carboxylase small chain
TBP: Tetrapyrrole-binding protein
TEM: Transmission electron microscope
Th: Thylakoid
