# Supplementary figures and images for: Soybean (Glycine max L. Merr.) seedlings response to shading: leaf structure, photosynthesis and proteomic analysis
Source: BMC Plant Biol. 2019 Jan 21;19:34. doi: 10.1186/s12870-019-1633-1 (PMC6341755; doi:10.1186/s12870-019-1633-1)

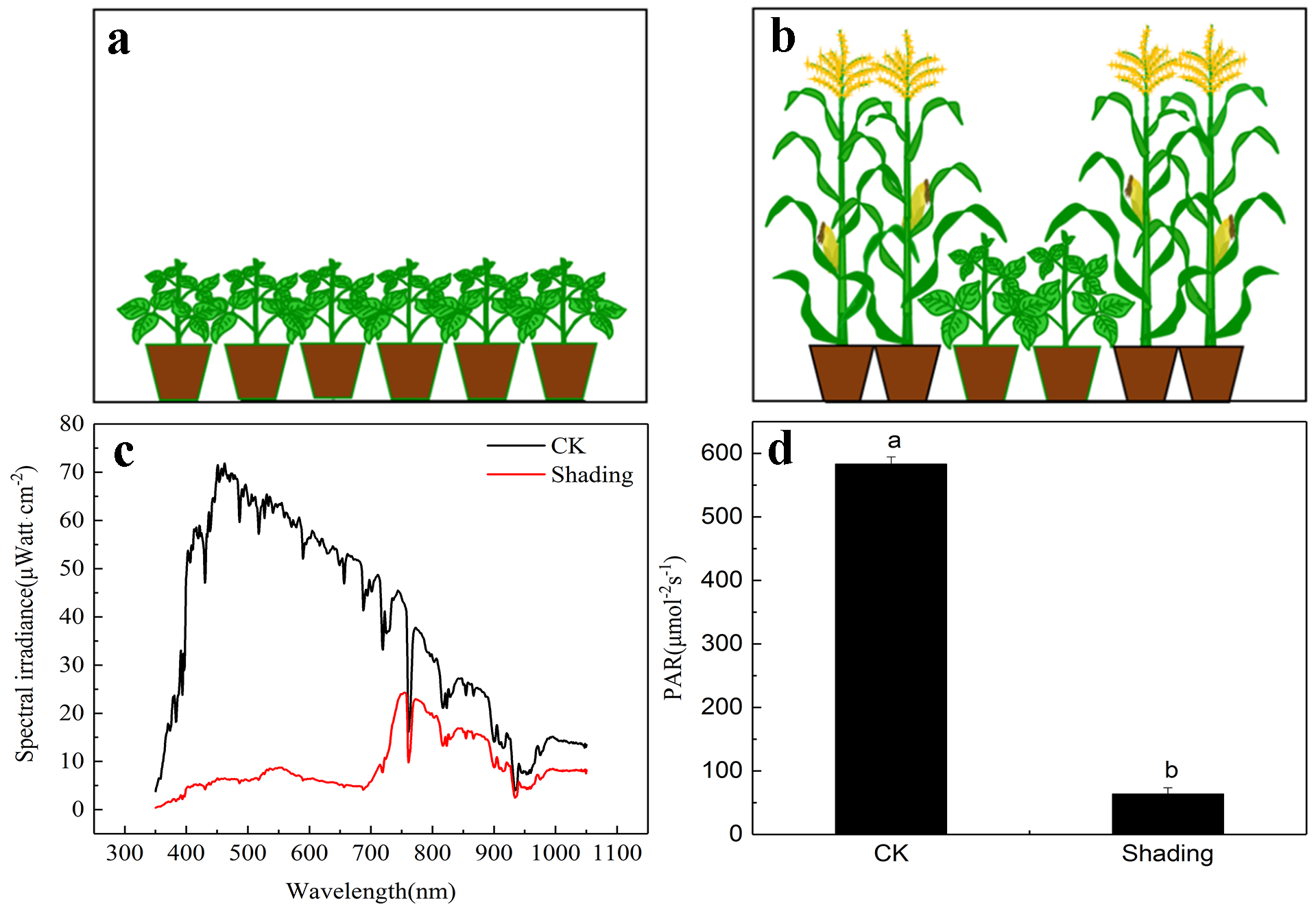

Supplement: Supplementary file 1 — Spectral irradiance (c) and PAR (d) of soybean canopy. a: normal light, b: maize–soybean relay strip intercropping. Significant differences between CK and shading treatments are indicated by different small letters (P < 0.05), respectively. (TIF 2260 kb) [file 12870_2019_1633_MOESM1_ESM.tif]

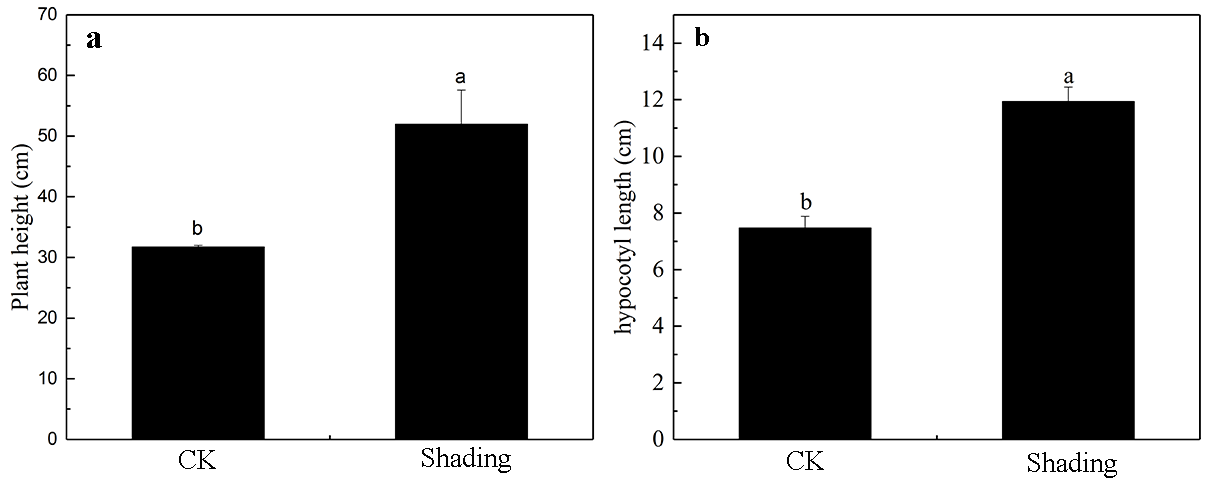

Supplement: Supplementary file 3 — Plant height (a) and hypocotyl length (b) of soybean plant. Significant differences between CK and shading treatments are indicated by different small letters (P < 0.05), respectively. (TIF 344 kb) [file 12870_2019_1633_MOESM3_ESM.tif]
