# Supplementary material for: Soybean (Glycine max L. Merr.) seedlings response to shading: leaf structure, photosynthesis and proteomic analysis
Source: BMC Plant Biol. 2019 Jan 21;19:34. doi: 10.1186/s12870-019-1633-1 (PMC6341755; doi:10.1186/s12870-019-1633-1)
Supplement: Supplementary file 2 — Real-time PCR primers. (PDF 309 kb) [file 12870_2019_1633_MOESM2_ESM.pdf]

Table S1. List of primers for characterizing Glycine max genes

**Gene Location (Annotation)**	**Protein Location**	**Forward primer**	**Reverse primer**
LOC100813807	A0A0R4J3L3	TGGCCTGCCGGGATTT	CAATGCCGGCAGATTTCG
LOC100779933(Gogh)	Q9XE94	AACTCCCCCTACGTTCTGCATT	GCTTCTCACCAACCCCATTG
LOC100784051	A0A0R0H273	TGACACTGAGATGGAGCAATACAAC	CATCATTGAGCTCCCAGATGA
LOC100799813	A0A0R4J5I3	CTGGTGAGCCCCCATCCTA	CAGCAGTGTCCCAACCATAGTC
LOC100793702(Cab-6)	Q93YG3	ATGGGACACTGCTGGACTATCA	CTCCAGCTCCCGGTTCCT
LOC100820335	I1MZ32	GGAGGTCGCCGACGTTAAA	ATCCCGAAAACCTCCGAGTAG
LOC100796767	I1M3X9	GCATTGAAGGAAGATCCAGAACA	ACTCGAGCCCACTCTTCCAA
LOC100526972	ILLA7	GGCATCAAGGGCACCAAAT	GCCTCCTCCAGCACTCACTATG
GLYMA_08G188500	A0A0R0IP95	ACCCCAGAGGGAGAGCAAGA	CTCTGCCTGGTCAAGAATGCA
